# Myasthenia Gravis Induced by Ipilimumab in a Patient With Metastatic Melanoma

**DOI:** 10.3389/fneur.2018.00150

**Published:** 2018-04-03

**Authors:** Vera Montes, Sandra Sousa, Fernando Pita, Rui Guerreiro, Cátia Carmona

**Affiliations:** Department of Neurology, Cascais Hospital Dr. José de Almeida, Lisboa, Portugal

**Keywords:** myasthenia, ipilimumab, immunity, safety, side effects

## Abstract

In daily clinical practice, there is a growing number of patients receiving new biological agents used in the treatment of malignancies. Ipilimumab is a fully humanized monoclonal antibody approved for patients with melanoma. It acts as an immune checkpoint inhibitor, binding and blocking cytotoxic T-lymphocyte antigen-4 in order to increase the antitumor immune response. There are several reports of autoimmune responses after its use. A 74-year-old man developed a mild rash and pruritus a few hours after the second infusion of ipilimumab and 24 h after the third dose of ipilimumab, he presented with shortness of breath, proximal limb muscle weakness, and diplopia. Repetitive nerve stimulation was consistent with a postsynaptic neuromuscular junction disorder. He began therapy with corticosteroids and pyridostigmine and ipilimumab was discontinued. Following ipilimumab suspension, the patient started to improve gradually. Here, we describe a rare case of myasthenia gravis presumably related with ipilimumab’s therapy. A better knowledge of these agents is necessary, in order to identify characteristics or biomarkers that may be associated with the development of potentially serious autoimmune responses.

## Introduction

Melanoma is the most aggressive skin tumor. In recent years, emergence of new immune-based molecularly targeted treatments dramatically improved the outcome of metastatic melanoma patients.

Ipilimumab is a fully humanized monoclonal antibody approved since 2011 for patients with unresectable or metastatic melanoma. It acts by direct blockade of the immune cytotoxic T-lymphocyte antigen-4 (CTLA-4), which is an inhibitor of T-cell activation, enhancing tumor-specific cellular immunity. This mechanism of action may lead to mild to moderate immune-related adverse effects (irAEs) and can involve the gastrointestinal tract, skin, and the endocrine and nervous systems (1, 2). Other immune checkpoint inhibitors, such as nivolumab, have also been associated with irAEs. Management guidelines have been developed and strongly advice initiation of corticosteroids in any patient in whom an irAE related to ipilimumab is suspected.

Autoimmune responses against nervous system have been described, such as myopathy, neuropathy, aseptic meningitis, and posterior reversible encephalopathy, but only three cases of myasthenia gravis (MG) have been reported in the medical literature (3, 4).

## Background and Case Presentation

Herein, we present a rare case of MG presumably related with ipilimumab’s therapy. A 74-year-old man, with history of hypertension and atrial fibrillation was diagnosed with metastatic melanoma in 2011. He started therapy with ipilimumab at a dose of 3 mg/kg every 3 weeks for a maximum of four doses. A few hours after the second infusion of ipilimumab, he developed a mild rash and pruritus. Physical examination at that time was unremarkable except for a mild macular rash.

Approximately 24 h after the third dose of ipilimumab, he presented with shortness of breath requiring oxygen supply, proximal limb muscle weakness, and binocular diplopia. Physical examination showed signs of respiratory distress, fatigable weakness, limitation of adduction of the right eye, and binocular diplopia.

**Figure 1:**
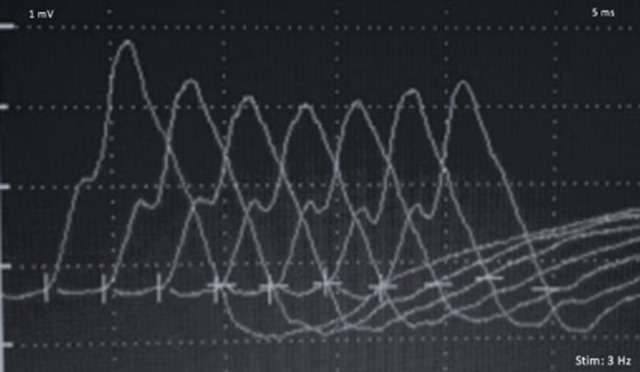
Repetitive nerve stimulation (3 Hz) with a 15% compound muscle action potential decrement, for the facial nerve.

Analytical study with thyroid-stimulating hormone and free thyroxine levels were normal. CT-brain and CSF analyses did not show alterations. Tensilon test was performed, showing a significant improvement of dyspnea and diplopia, measured qualitatively by the symptoms reported by the patient. Repetitive nerve stimulation was consistent with a postsynaptic neuromuscular junction disorder, with a 15% decrement at baseline for facial nerve (Figure 1). Acetylcholine receptor binding antibodies and Musk antibodies were negative. CT chest with contrast was revised and negative for thymoma. Ipilimumab was discontinued permanently and he began therapy with high-dose corticosteroids and pyridostigmine. Following ipilimumab suspension there was a marked improvement in the patient symptoms, and no further therapy with immunoglobulin or plasmapheresis was required.

The patient continued to improve gradually and after 1 month his only complain was diplopia; he had no complains of dyspnea and his muscular strength improved a lot, with capacity to autonomous walking. He is receiving a current corticosteroid dose of prednisone 40 mg per day and pyridostigmine 60 mg four times a day.

## Discussion

Ipilimumab has an antitumor response through blocage of CTLA-4, which normally downregulate immune response. Taking in consideration ipilimumab’s mechanism of action, it may induce a dysregulation of a preexisting immune response to self-antigens, which was held in check by CTLA-4. Nivolumab, another immune checkpoint inhibitor, has a resembling mechanisms of action, blocking programmed-cell death-1. This autoimmunity profile against normal self-tissues is most likely responsible for the irAEs that have been reported after the use of this type of immunotherapy. On the basis of these findings and given the absence of any possible etiology other than ipilimumab, we conclude that our patient had a MG secondary to ipilimumab. The lack of symptomatology prior to the use of the biological agent and the temporal relationship between the onset of myasthenic symptoms and drug administration, support our diagnostic hypothesis. Although most patients experience mild to moderate irAEs, a minority of patients may also experience severe, prolonged, and even irreversible adverse effects. Therefore, the absence of complete recovery does not exclude our diagnostic hypothesis. Taking into account the cellular mechanisms of action of CTLA-4, it is also expected not to find antibodies in MG induced by ipilimumab.

## Concluding Remarks

This clinical case highlights the importance of the recognition of adverse events, particularly neurological manifestations related to the activation of the immune system by ipilimumab. When early recognized and timely managed, most of these immune events are reversible, otherwise they can lead to severe or even life-threatening situations. Fatigable weakness, dyspnea, and vision disturbances are symptoms that may result from an autoimmune process directed against the nervous system and MG should be considered as a complication of therapy with CTLA-4 inhibitors. Clinicians should be aware of this toxicity profile, so as to promptly recognize, identify, and manage symptoms.

## Ethics Statement

Written informed consent was obtained from the participant for the publication of this case report.

## Author Contributions

VM: study concept and design, acquisition of data, analysis and interpretation of data, and drafting the manuscript. SS, RG, and CC: analysis and interpretation of data and critical revision of manuscript for intellectual content. FP: study supervision and critical revision of manuscript for intellectual content. The authors declare that they have each made substantial contributions to the conception, acquisition, analysis, and interpretation of the manuscript. All authors have critically revised the manuscript for intellectual content and have given their approval for the final version to be published.

## Conflict of Interest Statement

All authors declare that this work was conducted in the absence of any commercial or financial relationships.
